# Self-reported health, persistent symptoms, and daily activities 2 years after hospitalization for COVID-19

**DOI:** 10.3389/fncel.2024.1460119

**Published:** 2025-01-06

**Authors:** Roda Alhasan, Lena Rafsten, Alexandra C. Larsson, Katharina S. Sunnerhagen, Hanna C. Persson

**Affiliations:** ^1^Department of Clinical Neuroscience and Rehabilitation Medicine, Institute of Neuroscience and Physiology, Sahlgrenska Academy, University of Gothenburg, Gothenburg, Sweden; ^2^Department of Occupational Therapy and Physiotherapy, Sahlgrenska University Hospital, Gothenburg, Sweden; ^3^Department of Rehabilitation Medicine, Sahlgrenska University Hospital, Gothenburg, Sweden

**Keywords:** COVID-19, post Covid, persistent symptoms, respiratory symptoms, fatigue, shortness of breath, functional status, daily activities

## Abstract

**Introduction:**

Since the onset of the COVID-19 pandemic, 775 million cases have been reported globally. While many individuals recover fully, a significant proportion develop persistent symptoms. Numerous studies have investigated the long-term symptoms of COVID-19; however, the full extent and impact of these symptoms remain inadequately understood. The aim of this study was to investigate the prevalence of self-reported persistent symptoms, focusing on respiratory symptoms and fatigue and the impact on functional status 2 years after hospitalization for COVID-19.

**Methods:**

This study is prospective and includes participants from a longitudinal multi-center cohort that follows patients previously hospitalized due to COVID-19 (*n* = 211). The current study encompasses the 2-year follow-up, using post-hospitalization questionnaire surveys. Analyzed data were collected before discharge and at the 2-year follow-up. Participants were grouped by age, sex and COVID-19 severity and group comparisons where conducted. Logistic regression analysis was used to study functional impairment.

**Results:**

Two years after hospital discharge due to COVID-19, 125 participants completed the 2-year follow-up. The mean age of participants was 66 years (SD 12.2), and 68% were male. The majority of participants reported present respiratory symptoms (*n* = 83, 69%) and fatigue (*n* = 98, 78%) at the 2-year follow-up. Persistent respiratory symptoms and fatigue impacted functional status substantially (*p* = <0.001, *p* = 0.028, respectively). No significant differences were observed among groups depending on age, sex, or severity of COVID-19.

**Conclusion:**

For some individuals regardless of age, sex or COVID-19 severity, respiratory symptoms and fatigue may persist for up to 2 years following COVID-19. Hence, having available support from professionals knowledgeable about COVID-19 is imperative. Further research is important to unravel the mechanisms of long-term symptoms following COVID-19 and to develop effective therapeutic and rehabilitative interventions.

## 1 Introduction

Since the onset of the coronavirus disease 2019 (COVID-19) pandemic, over 775 million cases have been reported globally (last update 2024-06-16) (World Health Organization, 2023). While many patients recover from COVID-19 and return to their pre-COVID-19 health condition, a fair number develop persistent symptoms following acute COVID-19 (Ballering et al., 2022; Davis et al., 2023; Hanson et al., 2022; Yong, 2021; Tabacof et al., 2022). The World Health Organization (WHO) defines post COVID-19 as a medical condition that manifests in individuals who have a probable or confirmed post COVID-19 syndrome that characteristically emerges approximately 3 months after the onset of symptoms, persists for a minimum of 2 months and cannot be explained by an alternative diagnosis (World Health Organization, 2021). Estimates indicate that approximately 36 million individuals within the WHO European Region may have suffered from long COVID in the first 3 years of the pandemic (World Health Organization, 2023).

Respiratory symptoms and fatigue are commonly reported persistent symptoms after acute COVID-19 (Nalbandian et al., 2021; Katsarou et al., 2022; Huang et al., 2022; Townsend et al., 2020; Larsson et al., 2022; Fernández-de-las-Peñas et al., 2022; Munblit et al., 2021; Perlis et al., 2022). Prolonged symptoms following COVID-19 can negatively impact the ability to perform daily activities (Tabacof et al., 2022; World Health Organization, 2021; Larsson et al., 2022; Fernández-de-las-Peñas et al., 2022; Larsson et al., 2021; Palstam et al., 2024; Wahlgren et al., 2023). Previous studies suggested an association between female sex and occurrence of persistent symptoms within the first year following COVID-19 (Fernández-de-las-Peñas et al., 2022; Munblit et al., 2021; Perlis et al., 2022; Förster et al., 2022). Older age and severity of the initial COVID-19 have also been shown early after onset as possible risk factors for developing persistent symptoms following COVID-19 (Perlis et al., 2022; Förster et al., 2022). However, how these symptoms persist over time and which individuals previously hospitalized for COVID-19 are at higher risk for long-term symptoms post infection is still unclear. Therefore, following those patients is important for management and future medical consideration.

The International Classification of Functioning, Disability and Health (ICF) was developed by the WHO and can be used as a framework to ensure a multidimensional understanding of the consequences following COVID-19 (Vertaling van de WHO-Publicatie N, 2002).

The aim of the present study is to investigate the prevalence of self-reported persistent symptoms 2 years after hospitalization due to COVID-19, with a focus on respiratory symptoms and fatigue. Additionally, the study aims to assess the impact of respiratory symptoms and fatigue on functional status and to investigate differences based on sex, age, and initial COVID-19 severity.

## 2 Materials and methods

### 2.1 Study design and participants

This is a prospective study using data from the longitudinal multi-center cohort study, Life in the Time of COVID study in Gothenburg (GOT-LOCO) (Larsson et al., 2022; Larsson et al., 2021). Patients hospitalized for COVID-19 were consecutively recruited in the period from July 2020 through February 2021 from five hospitals in the Västra Götaland region, which has a population 1.67 million corresponding to 16% of the Swedish population. The inclusion criteria were patients who were admitted to a hospital within the region due to COVID-19, who were non-contagious when included in the study, who received hospital care for at least 5 days, were ≥18 years old, and who lived in the community prior to hospital admission. The exclusion criteria were inability to provide informed consent, expected survival <1 year (comorbidity indicating high mortality within 1 year, such as metastatic cancer or receiving palliative care), and patients who were not Swedish residents. Patients who fulfilled the inclusion criteria were included by the study coordinator or by a local test leader at each hospital prior to discharge. Patients were orally informed, and their written consent was obtained prior to the data collection procedure.

### 2.2 Data collection

Data before discharge were retrieved from medical charts and included COVID-19 severity and participants’ comorbidities. COVID-19 severity was assessed as either moderate or severe using the WHO clinical progression scale (Marshall et al., 2020). Participant’s comorbidities were classified using the ICD-10 version of the Charlson Comorbidity Index (CCI) from medical records (Charlson et al., 1987; Sundararajan et al., 2004). Age, sex, if treated in the intensive care unit (ICU) or not, total days in the ICU, and whether the participants were intubated were also included. The hospital length of stay (LOS) was defined by the number of days starting from the admission day to the discharge.

At this 2-year follow-up, a post survey consisting of different questionnaires was sent to each participant’s home. In case of no response, a text message with a reminder was sent to the participant. The participants were requested to return the survey in a prepaid reply envelope. If the participant had questions regarding the survey, a phone number for the research team was available.

The survey included general questions regarding the participant’s living situation, need for household help, medication, smoking status, height, weight and whether they have had a recurrence of COVID-19. Thereafter, questions regarding health status, physical activity (PA), and COVID-19 vaccination were included. Additionally, there were specific questions regarding symptoms related to COVID-19, the extent of the symptoms, difficulties in daily activities, and ongoing medical care or rehabilitation (including treatment by an occupational therapist, physical therapist, speech therapist, dietitian, or psychologist) due to COVID-19.

The survey also contained specific questionnaires to assess respiratory symptoms, fatigue, and the impact of COVID-19 on daily activities among the participants. The COPD Assessment Test (CAT) was used for self-assessment of respiratory symptoms, on an ordinal scale of 0–5 for each question and a total score of 40. A higher score indicates increased severity of respiratory symptoms (Jones et al., 2009). A Swedish version of the CAT was used. A cutoff value ≥10 was used to indicate presence of respiratory symptoms (Raghavan et al., 2012).

To assess fatigue, the Swedish version of the Multidimensional Fatigue Inventory (MFI-20) was used. The MFI-20 is a self-administered questionnaire that quantifies five different dimensions of fatigue: general fatigue, physical fatigue, decreased activity, decreased motivation, and mental fatigue, on an ordinal scale of 1–5 for each dimension of fatigue (Lundh Hagelin et al., 2007). A cutoff value ≥11 was used, as it exceeds the normative values for both males and females within the general population of Sweden (Engberg et al., 2017).

The Post-COVID-19 Functional Status scale (PCFS) was used for self-assessment of functional status 2 years after COVID-19 infection. The answers are graded on an ordinal scale of 0–4 where 0 indicates no limitations in daily activities (no functional impairment) and 4 indicates severe limitations in daily activities (severe functional impairment) (Klok et al., 2020). In this study, a cutoff value ≥1 is used to indicate impaired functional status.

The Saltin-Grimby Physical Activity Scale (SG-PALS) was used for self-assessment of the level of PA in participants. SG-PALS is a rating scale with four different elements ranging from 1 (sedentary lifestyle) to 4 (regular hard training) (Grimby et al., 2015). In the present study a cutoff value >1 is used to indicate that the participant is physically active.

### 2.3 Data handling and statistical analyses

All analyses were performed in the Statistical Package for the Social Sciences, IBM (SPSS) statistics, version 29. Descriptive statistics are used when presenting data. The groups were defined by sex (male or female), age (<65 years or ≥65 years), and COVID-19 severity (moderate or severe). The Mann–Whitney U test was used for group comparisons for variables with ordinal data. Dropout analysis was used to compare participants with nonparticipants regarding age, sex, level of medical care (ICU admission), and hospital LOS. The Mann–Whitney U test was used to analyze the variables age and hospital LOS, while Pearson’s chi-square test was used to analyze the variables sex and level of medical care.

Logistic regression analysis was used to define the association between functional status measured with PCFS considered as a dichotomous dependent variable. The continuous independent variables were age measured in years and hospital LOS measured in days. The categorical variables were sex, COVID-19 severity, CAT, MFI-20 (general fatigue), and SG-PALS at the 2-year follow-up. The PCFS, SG-PALS, CAT and MFI-20 questionnaires were dichotomized prior to the analysis. Cross tabs were used to ensure observations (>5) of each examined category.

The study used 95% confidence intervals (CIs). A *p*-value <0.05 was deemed statistically significant. If the CI for an odds ratio (OR) did not contain 1.0, the odds ratio was deemed statistically significant. This manuscript was prepared in accordance with the STROBE guidelines.

## 3 Results

### 3.1 Participants

**Figure 1:**
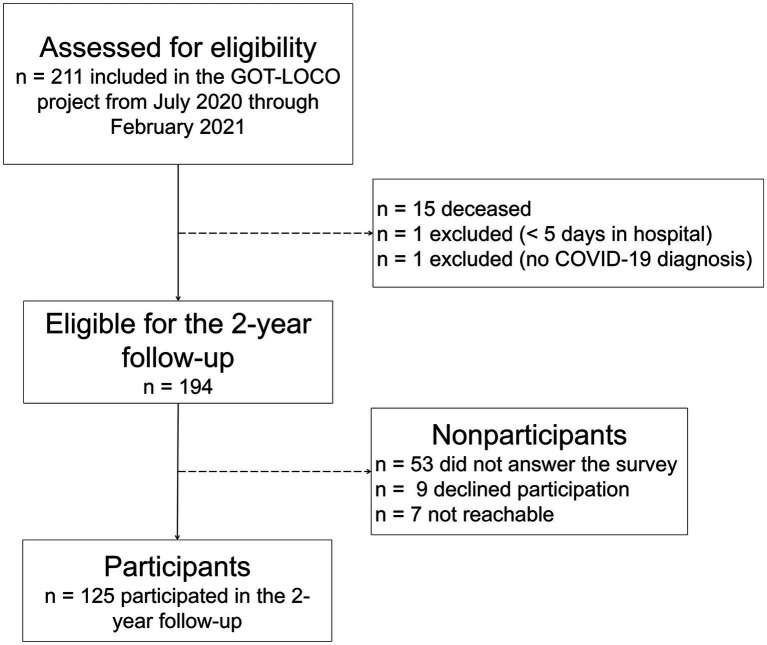
Flowchart of the inclusion of participants.

Of the 211 individuals who participated in GOT-LOCO, 194 were eligible for the 2-year follow-up, 178 of whom received the survey. The respond rate was 70%, with 125 participants completed the survey (Figure 1).

Nonparticipants had a significantly lower mean age than participants (*p* = 0.026). Regarding sex, level of medical care (ICU admission), and total days of hospital stay, no significant difference was found between participants and nonparticipants. Of the 125 participants, 85 (68%) were males and 72 (58%) were ≥65 years at the time of inclusion in the GOT-LOCO study. The mean hospital LOS was 32 (SD = 32.5) days (Table 1).

**Table 1:** Participant characteristics before discharge.

Variable	All participants (*n* = 125)	Sex		Age (years)		COVID-19 severity	
		Males (*n* = 85)	Females (*n* = 40)	<65 (*n* = 53)	≥65 (*n* = 72)	Moderate (*n* = 37)	Severe (*n* = 88)
Age, (y) mean (SD)	66 (12.2)	66 (10.4)	67 (15.3)	54 (7.6)	74 (6.8)	70 (13.1)	64 (11.5)
Sex, Males *n* (%)	85 (68)	–	–	37 (70)	48 (67)	20 (54)	65 (74)
Hospital LOS, (d) mean (SD)	32 (32.5)	37 (37)	21 (15.3)	32 (41.2)	32 (24.4)	13 (8.7)	40 (35.4)
ICU *n* (%)	64 (51)	49 (58)	15 (38)	31 (59)	33 (46)	0	64 (73)
Intubated *n* (%) (*n* = 121)	43 (36)	36 (43)	7 (18)	20 (39)	23 (33)	0	43 (51)
ICU LOS, (d) mean (SD)	19 (19.1)	21 (20.8)	12 (9.2)	19 (23.3)	19 (14.4)	–	19 (19.1)
CCI total score mean (SD) (*n* = 124)	1 (1.3)	1 (1.4)	1 (1.1)	1 (1.2)	1 (1.3)	1 (1.0)	1 (1.4)
CCI: Chronic pulmonary disease *n* (%) (*n* = 124)	40 (32)	26 (31)	14 (35)	12 (23)	28 (39)	9 (25)	31 (35)
CCI: Myocardial infarction *n* (%) (*n* = 124)	13 (11)	10 (12)	3 (8)	1 (2)	12 (17)	2 (6)	11 (13)
CCI: Heart failure *n* (%) (*n* = 124)	8 (7)	5 (6)	3 (8)	2 (4)	6 (8)	3 (8)	5 (6)
CCI: Diabetes *n* (%) (*n* = 124)	28 (23)	18 (21)	10 (25)	10 (19)	18 (25)	4 (11)	24 (27)
CCI: Malignancy *n* (%) (*n* = 124)	13 (11)	10 (12)	3 (8)	3 (6)	10 (14)	3 (8)	10 (11)
CCI: Cerebral vascular disease *n* (%) (*n* = 124)	5 (4)	4 (5)	1 (3)	2 (4)	3 (4)	1 (3)	4 (5)
CCI: Moderate to severe renal disease *n* (%) (*n* = 124)	15 (12)	13 (16)	2 (5)	7 (14)	8 (11)	1 (3)	14 (16)

Descriptive statistics were used to outline the participants’ characteristics. Data are presented as count = *n* and percentages (%), or mean and standard deviation (SD). Valid percentages are presented. The number of collected data = 125 unless another number is stated. LOS, length of stay; ICU, intensive care unit; CCI, Charlson comorbidity index.

At the 2-year follow-up, 45 (36%) of participants reported a need for household help, 77 (62%) lived with children or/and another adult, and 70 (56%) reported difficulties in daily activities (Table 2).

**Table 2:** Participant characteristics at the 2-year follow-up.

Variable	All participants (*n* = 125)	Sex		Age (years)		COVID-19 severity	
		Males (*n* = 85)	Females (*n* = 40)	<65 (*n* = 53)	≥65 (*n* = 72)	Moderate (*n* = 37)	Severe (*n* = 88)
Living situation							
Alone *n* (%)	48 (38)	32 (38)	16 (40)	11 (21)	37 (51)	21 (57)	27 (31)
With children and/or another adult *n* (%)	77 (62)	53 (62)	24 (60)	42 (79)	35 (49)	16 (43)	61 (69)
Need for household help *n* (%)	45 (36)	25 (29)	20 (50)	12 (23)	33 (46)	18 (49)	27 (31)
BMI mean (SD) (*n* = 104)	30 (6.5)	30 (5.6)	30 (8.3)	32 (6.9)	29 (6)	28 (5.4)	31 (6.8)
Smoking *n* (%) (*n* = 123)	1 (1)	0	1 (3)	0	1 (1)	1 (3)	0
Rehabilitation *n* (%) (*n* = 124)	21 (17)	13 (16)	8 (20)	11 (21)	10 (14)	5 (14)	16 (18)
Contact with healthcare *n* (%) (*n* = 123)	31 (25)	20 (24)	11 (28)	13 (25)	18 (25)	10 (28)	21 (24)
Recurrent COVID-19 *n* (%) *n* = 123	16 (13)	12 (15)	4 (10)	13 (25)	3 (4)	4 (11)	12 (14)
PA (*n* = 120)							
Sedentary *n* (%)	34 (28)	21 (26)	13 (33)	16 (31)	18 (26)	11 (31)	23 (27)
Active *n* (%)	86 (72)	60 (74)	26 (67)	35 (69)	51 (74)	24 (69)	62 (73)
Difficulties in daily activities *n* (%)	70 (56)	46 (54)	24 (60)	27 (51)	43 (60)	21 (57)	49 (56)

Descriptive statistics were used to outline the participants’ characteristics. Data are presented as count = *n* and percentages (%), or mean and standard deviation (SD). Valid percentages are presented. The number of collected data points = 125 unless another number is stated. BMI, body mass index; PA, physical activity.

### 3.2 Respiratory symptoms

**Figure 2:**
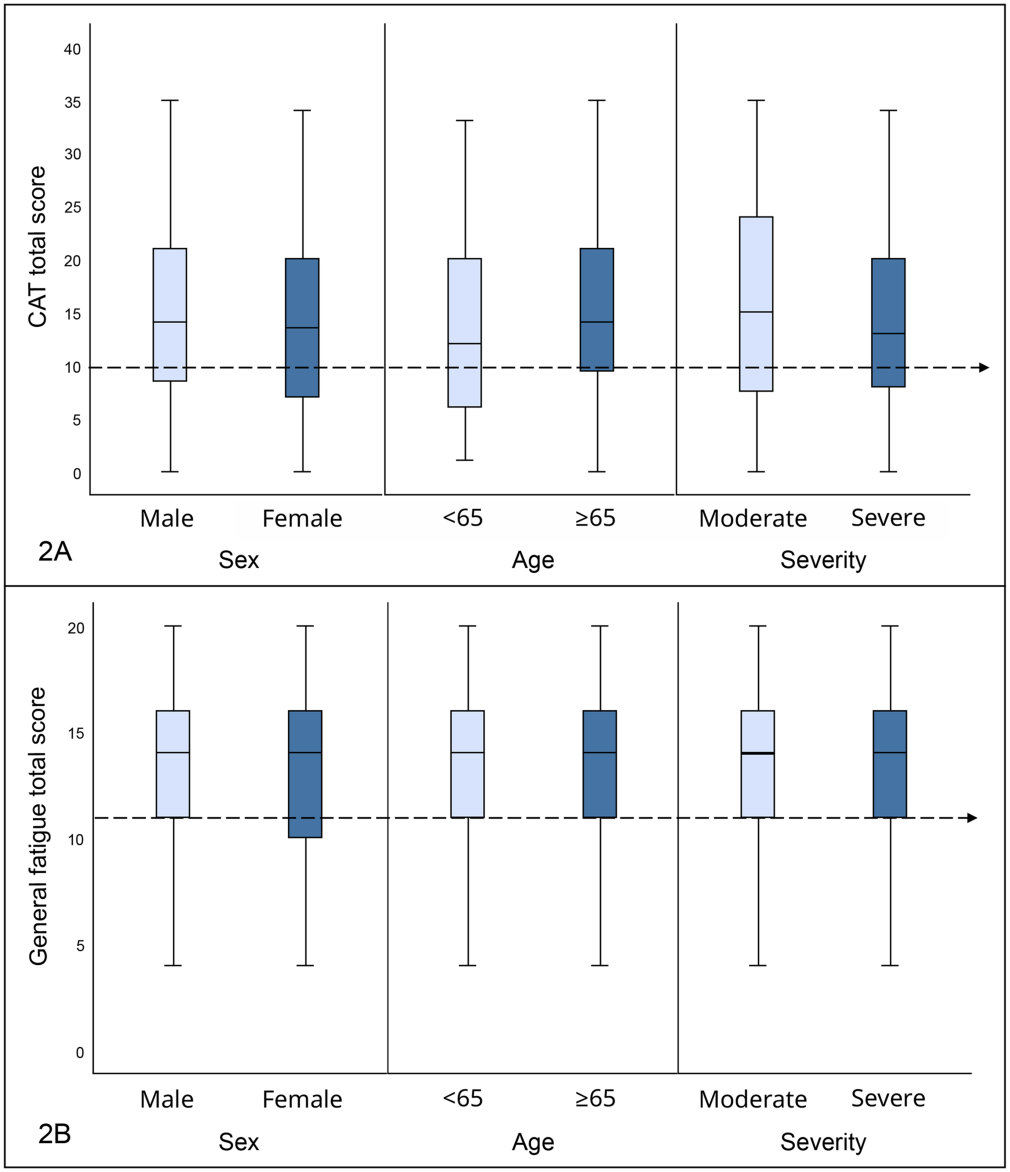
**(A)** Result of CAT depending on sex, age, and COVID-19 severity care presented in box plots. The total CAT score is presented on the y-axis, with a range of 0–40 points. The cutoff value is marked by a horizontal dashed line. The different groups defined by sex (male, female), age (<65, ≥65 years) and COVID-19 severity (moderate, severe) are presented on the x-axis. CAT, COPD Assessment test. **(B)** Result of general fatigue measured with the multidimensional fatigue inventory inventory-20 (MFI-20) depending on sex, age, and COVID-19 severity presented in box plots. Total score of general fatigue is presented on the y-axis, with a range of 0–20 points. The cutoff value is marked by a horizontal dashed line. The different groups defined by sex (male, female), age (<65, ≥65 years), and COVID-19 severity (moderate, severe) are presented on the x-axis.

Approximately 69% of participants (*n* = 83) reported respiratory symptoms (CAT total score ≥ 10). Additionally, the observed median of the total CAT score among participants indicated the presence of respiratory symptoms among all groups divided on age, sex, and COVID-19 severity, however, there were no significant differences between groups (Figure 2;Table 3).

**Table 3:** Outcomes regarding respiratory symptoms, fatigue, and functional status after COVID-19 at the 2-year follow-up.

Variable	All participants (*n* = 125)	Sex		*p*	Age (years)		*p*	COVID-19 severity		*p*
		Males (*n* = 85)	Females (*n* = 40)		<65 (*n* = 53)	≥65 (*n* = 72)		Moderate (*n* = 37)	Severe (*n* = 88)	
CAT median (IQR) (*n* = 121)	14 (13)	14 (12)	14 (15)	0.619	13 (15)	14 (11)	0.327	15 (17)	13 (12)	0.428
MFI-20										
General fatigue median (IQR) (*n* = 125)	14 (5)	13 (5)	14 (4)	0.720	14 (5)	14 (5)	0.998	14 (5)	14 (5)	0.637
Physical fatigue median (IQR) (*n* = 124)	15 (6)	14 (7)	16 (5)	0.429	15 (6)	15 (6)	0.798	15 (5)	15 (6)	0.506
Mental fatigue median (IQR) (*n* = 119)	11 (8)	11 (7)	10 (10)	0.411	11 (5)	10 (9)	0.496	11 (7)	10 (8)	0.145
Reduced motivation median (IQR) (*n* = 123)	10 (6)	10 (6)	10 (6)	0.326	10 (6)	11 (5)	0.121	11 (5)	10 (6)	0.219
Reduced activity median (IQR) (*n* = 124)	13 (5)	13 (5)	13 (5)	0.857	13 (5)	13 (5)	0.211	13 (5)	13 (5)	0.915
PCFS median (IQR) (*n* = 106)	1 (2)	1 (2)	2 (2)	0.382	1 (2)	1 (3)	0.392	2 (3)	1 (2)	0.200
SG-PALS median (IQR) (*n* = 120)	2 (2)	2 (2)	2 (1)	0.073	2 (1)	2 (2)	0.420	2 (1)	2 (2)	0.181

Descriptive statistics were used. Data are presented as count = *n*, percentages (%), or median and interquartile range (IQR). The Mann–Whitney U test was used for group comparisons. *p*, *p*-value; CAT, COPD Assessment Test; MFI-20, Multidimensional Fatigue Inventory; PCFS, Post-COVID Functional Status Scale; SG-PALS, Saltin-Grimby Physical Activity Level Scale.

**Figure 3:**
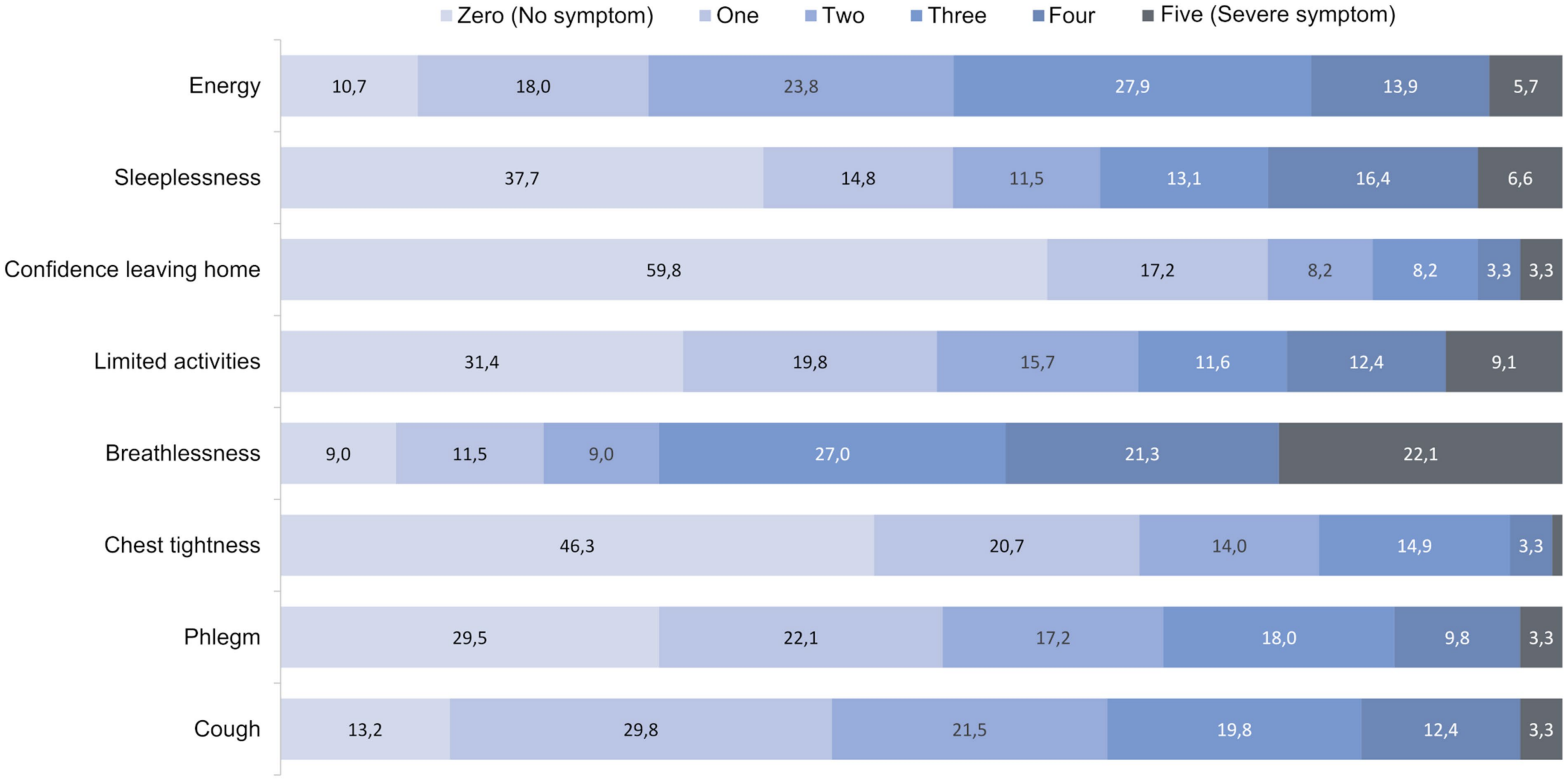
Distribution of the eight different elements of CAT in percentages ranging from zero = 0 points (no symptom) to five = 5 points (severe symptom). The eight different elements are presented on the x-axis, while the total points are represented in the stacked bars on the y-axis. Values below 1% are not labeled. CAT, COPD Assessment Test.

The most common respiratory symptom reported by participants was breathlessness (Figure 3), with 22% reporting severe symptoms. The presence of cough and an impact on energy level were also commonly reported (Figure 3).

### 3.3 Fatigue

Approximately 78% (*n* = 98) of participants reported general fatigue, 77% (*n* = 96) reported physical fatigue, and 74% (*n* = 92) reported reduced activity. Additionally, general fatigue, physical fatigue, and reduced activity assessed with the MFI-20 had elevated medians among all groups based on age, sex and COVID-19 severity (cutoff ≥11) (Figure 2;Table 3). The median values of mental fatigue and reduced motivation bordered on elevated or were slightly under the normative value. No significant difference in fatigue depending on age, sex, or COVID-19 severity was detected (Table 3).

### 3.4 PA and functional status

About 72% (*n* = 86) of participants reported being physically active 2 years after hospitalization due to COVID-19, while about 28% (*n* = 34) had a sedentary lifestyle (Table 2). At the 2-year follow-up, no significant difference in PA was found between groups based on age, sex, or COVID-19 severity (Table 3).

Approximately 45% of the participants reported no functional impairment (*n* = 40) or negligible functional impairment (*n* = 16) on PCFS. In contrast, approximately 40% reported mild to severe functional impairment. A negative impact on functional status was noted in all groups (PCSF) but no significant difference was found between groups based on age, sex, or COVID-19 severity (Table 3).

### 3.5 Impact of age, sex, and level of medical care on functional status

Two separate logistic regression analyses were performed to study functional impairment 2 years after COVID-19. The first regression analysis model included factors from the acute care phase before discharge from hospital. No significant association was noted between functional impairment and any of the factors before discharge (Table 4).

**Table 4:** Multivariable logistic regression predicting the likelihood of functional impairment occurrence and association with age at admission, sex, COVID-19 severity, and total number of days in hospital.

Variables		OR	*p*-value	95% CI for EXP (B)	
				Lower	Upper
Age (years)		1.019	0.289	0.984	1.055
Sex	Males	Reference	Reference	0.535	3.353
	Females	1.339	0.533		
COVID-19 severity	Moderate	Reference	Reference	0.237	1.600
	Severe	0.616	0.320		
Hospital LOS (days)		1.006	0.388	0.993	1.019

Logistic regression analysis was used. OR, odds ratio; CI, confidence interval; LOS, length of stay.

The second logistic regression model included factors from the 2-year follow-up. A significant association was found between functional impairment and respiratory symptoms measured with CAT and general fatigue measured with the MFI-20 (Table 5). No significant association was found between functional impairment and age or level of PA at the 2-year follow-up (Table 5).

**Table 5:** Multivariable logistic regression predicting the likelihood of functional impairment occurrence and association with age at 2-year follow-up, total CAT score, total general fatigue score, and PA at 2-year follow-up.

Variables		OR	*p*-value	95% CI for EXP (B)	
		OR	*p*-value	Lower	Upper
Age (years)		1.021	0.330	0.979	1.066
CAT	<10	Reference	Reference	3.277	28.894
	≥10	9.731	<0.001		
MFI-20 (general fatigue)	<11	Reference	Reference	1.163	15.134
	≥11	4.195	0.028		
SG-PALS	Sedentary	Reference	Reference	0.148	1.776
	Active	0.512	0.291		

Logistics regression analysis was used. OR, odds ratio; CI, confidence interval; CAT, COPD Assessment Test; MFI-20, Multidimensional Fatigue Inventory; SG-PALS, Saltin-Grimby Physical Activity Scale.

## 4 Discussion

Two years after hospital care due to COVID-19, respiratory symptoms were commonly reported in people that previously received hospital care. For some participants, self-reported respiratory symptoms were present for up to 2 years after hospitalization for COVID-19, however, there were no substantial differences depending on age, COVID-19 severity, or sex. Recently, it was shown that during the pandemic the age standardized mortality rate in Sweden was low compared to other European countries (Pizzato et al., 2024), this is in contrast to the beginning of the pandemic, when Sweden had an excess mortality and less intense government restrictions were implemented (Lam et al., 2009; Ludvigsson, 2023). Despite the mortality rate, only small functional outcome differences depending on ICU treatment in the first or second/third wave of the pandemic was shown in a Swedish population (Darlington et al., 2023). On the other hand, the need for specialized rehabilitation was less in the second wave, compared to the first wave, although rehabilitation was beneficial for all admitted patients (Gabunia et al., 2023). This might indicate functional outcome differences in different stages of the COVID-19 pandemic and between countries. The present study comprises a multi-center cohort from the early phase (first and second wave in Sweden) of the pandemic, including a 2 year follow up.

The large prevalence of respiratory symptoms in the present study may be influenced by our study population having moderate to severe COVID-19, with all participants receiving respiratory support (oxygen supplement, intubation, or mechanical respirational support) during their hospitalization. In addition, about one of three participants suffered from chronic pulmonary disease prior to hospitalization for COVID-19, which may impact the prevalence of respiratory symptoms. Previous research has shown that respiratory symptoms can linger following COVID-19 (Katsarou et al., 2022; Huang et al., 2022; Larsson et al., 2022; Fernández-de-las-Peñas et al., 2022). Respiratory assessments by physiotherapists specialized in respiratory care and effective self-management may help to improve the burden of persistent respiratory symptoms (Fagevik Olsén et al., 2024). Additionally, respiratory muscle training has been shown to be beneficial (McNarry et al., 2022; Fagevik Olsén et al., 2024).

Regarding the MFI-20 domains of general fatigue, physical fatigue, and reduced activity, the findings of the current 2-year follow-up were considerably higher when compared with normative values from the Swedish population (Fernández-de-las-Peñas et al., 2022; Engberg et al., 2017). Nearly 4 out of 5 participants reported general fatigue, physical fatigue, and reduced activity. No notable impact was present in the domains of mental fatigue and reduced motivation. Other studies have previously reported fatigue as a common persistent symptom after COVID-19 (Katsarou et al., 2022; Huang et al., 2022; Townsend et al., 2020; Larsson et al., 2022). Additionally, fatigue may persist following viral infections in certain individuals, as evidenced by chronic fatigue persisting for up to 4 years following severe acute respiratory syndrome, in accord with our research findings (Lam et al., 2009). However, the fact that mental fatigue and reduced motivation in the present study fall within normal ranges suggests that fatigue and its impact on the participants may persist regardless of personal motivation and mental tiredness. The normal score in the reduced motivation domain may be explained by the motivation to regain normal life after illness and hospitalization (Törnbom et al., 2022). In the present study, no significant difference in fatigue outcomes was found among groups based on age, sex, or COVID-19 severity. However, our findings conflict with earlier suggestions that females have, in general, higher fatigue levels in comparison with men (Engberg et al., 2017). Additionally, previous studies suggest that females experience persistent fatigue after COVID-19 more than males (Townsend et al., 2020; Fernández-de-las-Peñas et al., 2022).

The self-reported PA 2 years after the initial COVID-19 demonstrated that most participants were physically active. The relatively high rate of individuals assessing themselves as physically active may be a result of the rehabilitation gained due to functional impairment following the hospitalization (Larsson et al., 2021). The participants may be influenced by being critically ill or hospitalized for COVID-19 and thus gained motivation to engage in PA in order to regain their strength and ordinary level of activity (Engwall et al., 2022). No significant differences regarding PA were found among groups defined by age, sex, and COVID-19 severity, which conflicts with previous studies that found differences in PA based on level of medical care and sex (Larsson et al., 2022; Engberg et al., 2017). Hence, although differences between groups may be present at the time of recovery from acute COVID-19 and in the first period after recovery, they may diminish over time. Although most participants reported some PA in their daily life, they were less active than the working-age population (mean age = 51 years) in a previous study that was conducted 18 months post COVID-19 (Palstam et al., 2024). This suggests a need for additional support and rehabilitation to enhance PA outcomes in older individuals, as participants in the present study were generally older (mean age = 66 years).

Moreover, our results revealed an overall functional impairment among participants when measured with the PCFS. The majority of cases of severe functional impairment were among older participants. However, due to the low frequency of the severe functional impairments that were reported, no definitive conclusions can be established. These results may be influenced by comorbidities or challenges in rehabilitating functional impairment at older age. A notable proportion of participants reported a need for household help and difficulties in performing daily activities, potentially due to functional impairment following COVID-19 complications. A considerable number of participants lived with another adult or/and children, suggesting they may receive help with daily activities from next of kin, complicating a comprehensive assessment of their functional status. Our findings correspond to previous studies that noted limitations in daily activities following COVID-19 (Fernández-de-las-Peñas et al., 2022; Palstam et al., 2024; Wahlgren et al., 2023). Additionally, a significant association between functional impairment and the presence of respiratory symptoms and general fatigue was noted, which emphasizes the impact of these symptoms on functional status. However, no association between functional impairment and age, sex, COVID-19 severity, hospital LOS, or the level of PA at 2 years after COVID-19 was found. When comparing functional status among groups defined by age, sex, and COVID-19 severity, no considerable differences were noted. This finding indicates that suitable medical care should be offered to all patients with persisting symptoms regardless of sex, age or COVID-19 severity.

The present study followed a well-defined population from multiple centers over a long time. The participants were examined early in the disease process (hospital, first and second wave of the pandemic), allowing a detailed understanding of the study population’s characteristics in the acute setting. Although, the current follow-up occurred 2 years after discharge, a response rate of over 70% was registered. The questionnaires were well-structured and quantitative, which reduces measurement bias and increases credibility. A phone number was provided to the participants to answer any questions they had, which decreased the risk of misunderstanding. The age of nonparticipants was considerably lower than the age of participants. This might have many possible explanations, such as perceived improvement in their health status, loss of motivation due to absence of improvement or lack of time. These are questions that remains to be answered.

Our study has certain limitations. As the data were self-reported, there is risk of reporting bias, which can be influenced by many personal factors. Additionally, the absence of a control group complicates charting the occurrence and burden of symptoms in comparison with other individuals. Testing for COVID-19 was not fully established at the beginning of the study, and this can be considered a limitation. Given that the present study focused on a multi-center cohort from Swedish hospitals during the early stages of the COVID-19 pandemic, and recognizing Sweden’s unique response to the pandemic resulting in divergent treatment and management strategies compared with those of other countries, it is important to interpret the results with some caution when extrapolating our findings to broader populations of COVID-19 patients worldwide (Ludvigsson, 2023).

## 5 Conclusion

Respiratory symptoms and fatigue can persist for 2 years after acute COVID-19 which can lead to functional impairment that discourages participation in daily activities. Personal factors such as age, sex, and COVID-19 severity may be inadequate as predictors of risk for persistent COVID symptoms 2 years after hospitalization. Therefore, it is important to offer follow-up as well as customized care to this group of patients regardless of age, sex, and COVID-19 severity. More research is essential to further understand the pathophysiology of long-term consequences of COVID-19 and develop therapeutic approaches and rehabilitation methods that may improve the prognosis of patients with persistent symptoms following COVID-19.

## Data Availability

The raw data supporting the conclusions of this article will be made available by the authors without undue reservation.
